# Brazilian malaria molecular targets (BraMMT): selected receptors for virtual high-throughput screening experiments

**DOI:** 10.1590/0074-02760180465

**Published:** 2019-02-25

**Authors:** Renata Rachide Nunes, Amanda Luisa da Fonseca, Ana Claudia de Souza Pinto, Eduardo Habib Bechelane Maia, Alisson Marques da Silva, Fernando de Pilla Varotti, Alex Gutterres Taranto

**Affiliations:** 1 Universidade Federal de São João Del-Rei, Divinópolis, MG, Brasil; 2 Centro Federal de Educação Tecnológica de Minas Gerais, Divinópolis, MG, Brasil

**Keywords:** docking, virtual screening, structure based drug design and bioinformatics

## Abstract

**BACKGROUND:**

Owing to increased spending on pharmaceuticals since 2010, discussions about rising costs for the development of new medical technologies have been focused on the pharmaceutical industry. Computational techniques have been developed to reduce costs associated with new drug development. Among these techniques, virtual high-throughput screening (vHTS) can contribute to the drug discovery process by providing tools to search for new drugs with the ability to bind a specific molecular target.

**OBJECTIVES:**

In this context, Brazilian malaria molecular targets (BraMMT) was generated to execute vHTS experiments on selected molecular targets of *Plasmodium falciparum*.

**METHODS:**

In this study, 35 molecular targets of *P. falciparum* were built and evaluated against known antimalarial compounds.

**FINDINGS:**

As a result, it could predict the correct molecular target of market drugs, such as artemisinin. In addition, our findings suggested a new pharmacological mechanism for quinine, which includes inhibition of falcipain-II and a potential new antimalarial candidate, clioquinol.

**MAIN CONCLUSIONS:**

The BraMMT is available to perform vHTS experiments using OCTOPUS or *Raccoon* software to improve the search for new antimalarial compounds. It can be retrieved from www.drugdiscovery.com.br or download of Supplementary data.

Innovations in health science have resulted in drastic changes in the ability to treat disease, improving the quality of human life. Since the late 1990s, pharmaceutical spending has grown faster than other major components of the health care system. Consequently, discussions on the increased costs to develop new medical technologies have been increasingly focused on the pharmaceutical industry.

Among new technologies, bioinformatics tools have been highlighted with the creation of several data banks. These data banks have contributed to the drug discovery process providing tools to search for new drugs able to bind to specific molecular targets. The molecular target is often associated with enzymes, receptors, or other proteins that can be altered by an external stimulus.^1^ In this context, banks such as the Protein Data Bank (PDB), Tropical Disease Research (TDR), ChEMBL, ZINC database, and PlasmoDB are dedicated to describing the molecular target information. In contrast, the Zinc database, DrugBank, and PubChem are addressing to the ligand information. The ChEMBL provides information about targets and ligands and their integration with each other. PDB is a data bank with more than 100,000 macromolecules, including proteins and nucleic acids. The TDR database (http://tdrtargets.org) is an on-line resource to facilitate the identification and selection of molecular targets for drug development, focusing on pathogens responsible for neglected human diseases.^2^ Similarly, PlasmoDB is a database that provides genetic and functional information, as well as evidence of transcription, proteomics, protein function, and evolution. Furthermore, ligands can be obtained from databases in different file formats, such as mol2 and PDB, or their 3D structures can be generated using programs and databases, such as MarvinSketch, ZINC database, GaussView, Molden, Drugbank, and PubChem.

Many drugs studied in clinical trials fail and are not approved for use in humans. An alternative to overcome this situation is the use of computer-assisted drug design (CADD) for rational development of new drugs.^3^ Molecular modeling techniques have been used in the discovery of novel drugs as their applications have shown efficiency in several stages of the rational planning process.^4^ CADD uses many computational techniques to discover, design, and search for biologically active compounds.

Through computer methods, pharmaceutical companies have introduced new drugs in the market. Among the most successful cases are the development of imunodeficiency virus (HIV) protease inhibitors.^5^ HIV-1 proteases inhibitors were identified using techniques such as pharmacophore standard molecular docking and molecular dynamics (MD), which is a structure-based drug design approach (SBDD). Another successful case was the development of sialidase enzyme inhibitors of the influenza virus, which were identified by rational design based on the crystallographic structure of the enzyme. These studies led to the release of Zanamivir, an Food and Drug Administration (FDA)-approved drug in 1999, used to treat infections caused by influenza viruses.^6^ In addition, potent inhibitors for the treatment of tuberculosis were discovered using ligand-based drug design (LBDD) and MD methods.

These exemplars show that CADD methods are useful to identify and select molecules able to interact with high affinity and selectivity against a specific molecular target. In summary, two strategies have been used in CADD projects, LBDD and SBDD. As both strategies require an extensive discussion, only SBDD will be highlighted in this manuscript. Among SBDD methods, molecular docking, highlighted by its practice, is fast and has low computation cost. The docking between ligand and the molecular target is the first step in any cell signaling pathway.^7^ In this context, programs are used to insert the ligand into the target binding site to find the best conformation, predicting the intermolecular interaction between the ligand-receptor and estimating its affinity.^8^ The docking method can be applied to hundred or thousand compounds against a specific molecular target. In this study, docking is referred to as target-based virtual screening (TBVS). In addition, TBVS can be evaluated against several molecular target data banks to predict the molecular target of ligands, similar to high-throughput screening (HTS) experiments. Virtual screening (VS) became widely used in 1990 during the initial phases of research into the drug discovery process for the identification and selection of new bioactive molecules.^9^ In this study, VS is denoted as virtual high-throughput screening (vHTS). Specifically, vHTS is a computational method of molecular docking to predict the molecular target of ligands by estimating their binding affinity.^10^ ^,^ ^11^ Therefore, vHTS can enrich the ligand database in HTS studies, reducing the cost of the drug discovery process. Even though genomics, proteomics, and ligands databanks have been established, the platform to perform automated TBVS is still unknown, including for neglected diseases. The OCTOPUS platform^12^ was developed to optimise the molecular docking process. As such, we were motivated to build the Brazilian malaria molecular targets (BraMMT) database. Malaria is one of the world’s most serious public health problems.^13^ Therefore, it is necessary to develop new therapeutic alternatives to improve the existing chemotherapy. In this context, BraMMT is composed of *Plasmodium falciparum* molecular targets retrieved from the PDB. This data bank permits *in silico* vHTS experiments against a pool of *P. falciparum* molecular targets. In this paper, the BraMMT was evaluated through docking tools and a set of known antimalarial compounds.

## MATERIALS AND METHODS

*Selection and preparation of molecular targets* - The three-dimensional structures of the receptors were obtained from the PDB database from their respective codes^14^ using the key word: *Plasmodium falciparum*. For the molecular target with several entries in the PDB, the structure with the lowest values crystallographic resolution was considered, which had the ligand in the binding site. In addition, the molecular targets *P. falciparum* ATPase calcium pump ortholog (PfATP6) and hexose transporter *P. falciparum* (PfHT) were constructed by comparative modeling.^15^ ^,^ ^16^

Thereafter, the molecular targets were prepared by removing the replicate residue present at the binding site. Moreover, only water molecules that carried out at least two interactions between the ligand and molecular target were kept.^7^ ^,^ ^10^ Further, the protonation state of each target was adjusted according to the pH of the enzymatic environment using the PROPKA module (academic version) of the Maestro software.

Finally, the druggability of each target was evaluated by TDR platform targets (http://tdrtargets.org). This characteristic predicts whether a protein can bind with high affinity and specificity to small compounds.

*Evaluation of molecular docking* - Re-docking methodology was carried out to evaluate the AutoDock Vina program.^11^ ^,^ ^17^ All calculations were made in triplicate and expressed as the mean. For each target, the AutoDock Tools program was used to obtain the 20 Å boxes and the x, y, and z coordinates, with spaced points of 1 Å centered on the ligand. In addition, the crystallographic structures without ligand, a search for equivalent structure belongs to another organism, was performed using the BLAST program. The degree of identity was greater than 27%, which is considered satisfactory to use the active compounds belonging to the target in another organism.^18^ Hence, the atomic molecular coordinates of the ligand were transferred from the structure found by BLAST to the *P. falciparum* structure following a re-docking process. The crystallographic and re-docking ligands were overlaid for calculation of root mean square deviation (RMSD) using the Discovery Visualizer 4.5 program.

Additionally, the receiver-operator characteristic (ROC curve) and the area under the ROC curve (AUC) were established for each molecular target to evaluate the ability of the molecular docking methodology to differentiate the active molecules from decoys (false positives).^19^ For each molecular target from BraMMT, at least two active compounds with the lowest Ki or IC_50_ value were selected from ChEMBL.^20^ Subsequently, inactive compounds (decoys) were obtained from the active compounds for each molecular target using the DUD-E platform. Decoys had similar physical properties, such as molecular mass, number of rotational bonds, Log P, and number of hydrogen bond donor/hydrogen bond acceptor groups. Following, the curves, ROC and AUC, were built using SPSS Statistics for Windows software.

Active compounds and decoys were submitted to the molecular docking calculations in the AutoDock Vina program^11^ ^,^ ^17^ using OCTOPUS,^12^ in which the configuration files were determined through a re-docking step.

*Virtual screening of antimalarial drugs* - The BraMMT data bank was evaluated using 27 antimalarial drugs [see Supplementary data (Table V)] listed by the World Health Organization (WHO) (https://www.ebi.ac.uk/chembl/malaria/drugstor). These molecules were selected from the ChEMBL platform for TBVS through OCTOPUS,^12^ maintaining the parameters used in the molecular re-docking step [see Supplementary data (Table I)].

Finally, the 27 antimalarial drugs were ranked using the Equation 1, which ∆ values were obtained by the difference between the crystallographic ligand binding energy (obtained from the re-docking step) and antimalarial drugs binding energy (obtained from the vHTS process). Thus, the Δ values greater than 0 demonstrated that the respective compound had a higher binding energy than the crystallographic ligand; therefore, it could be recognised by the molecular target through the intermolecular interaction.

∆ = (crystallographic ligand energy - antimalarial drugs energy)

Equation 1. Δ values obtained from the difference between binding energies of the crystallographic ligand and the antimalarial drugs.

*Molecular dynamics* - The molecular dynamics of selected compounds were performed by MolAr in-house software. MolAr was used to refine ligands through MOPAC2016 using the Parametric Method 7 (PM7) and EF routine to search for the structure of local minimum and to perform a molecular dynamic using DOCK 6. In the simulations, ligand flexibility was recorded. Flexible simulations were performed in 3000 steps and 100 energy minimisation cycles.

*Chemicals* - Clioquinol (5-Chloro-7-iodo-8-quinolinol, 5-Chloro-8-hydroxy-7-iodoquinoline, Clioquinol, Iodochlorhydroxyquin) was purchased from Sigma (CAS Number 130-26-7). Chloroquine diphosphate salt (N4-(7-Chloro-4-quinolinyl)-N1,N1-dimethyl-1,4-pentanediamine diphosphate salt) was purchased from Sigma (CAS Number: 50-63-5).

*In vitro schizonticidal activity of the clioquinol against P. falciparum* - *P. falciparum* chloroquine-resistant (W2) strain were maintained in continuous culture using human red blood cells in RPMI 1640 medium supplemented with human plasma. Human red blood cells and human plasma were provided by Foundation of Hemotherapy and Hematology of Minas Gerais (Fundação Hemominas). The parasites were synchronised using sorbitol treatment, and the parasitaemias were evaluated microscopically with Giemsa-stained blood smears. The antimalarial activity was determined using an enzyme-linked immunosorbent assay (ELISA) antiHRPII assay. Infected red blood cells were plated in a 96-well plate at 0.05% parasitaemia and 1.5% haematocrit. Different concentrations of the clioquinol were added in triplicate, and twelve drug-free wells were used as controls (six frozen after 24 h as the HRPII background). After the incubation (72 h), the plate was frozen and thawed twice, and an ELISA using antiHRPII antibodies was performed.

The results were expressed as the mean of the half-maximal inhibitory dose (IC_50_) of three assays performed in triplicate, compared with drug-free controls. Curve fitting was performed using OriginPro 8.0 software (Origin Lab. Corporation, Northampton, MA, USA).

*In vitro cytotoxicity test* - The noncancerous human lung fibroblast cell line WI-26-VA4 (ATCC CCL-95.1) was used to assess the cell viability after each chemical treatment employing the MTT colorimetric assay. Briefly, 1 × 10^6^ cells were plated in 96-well microplates with RPMI 1640 medium supplemented with foetal bovine serum (FBS). Then, microplates were incubated overnight at 37ºC, 5% CO_2_, followed by the treatment with each compound solubilised in DMSO 0.1% (v/v). Negative control groups were constituted of cells without treatment. Five serial dilutions (1:10) were made from a stock solution (10 mg·mL^-1^) using RPMI supplemented with 1% FBS. After 48 h of incubation, cell viability was evaluated by discarding the medium and adding 100 μL of MTT 5%, followed by 3 h of incubation. Then, the supernatant was discarded, and the insoluble formazan product was dissolved in DMSO. The optical density (OD) of each well was measured using a microplate spectrophotometer at 550 nm. The OD in untreated control cells was defined as 100% cell viability. All assays were performed in triplicate. The SI of clioquinol was calculated as: SI = IC_50_ WI-26-VA4 / IC_50_ *P. falciparum*.

## RESULTS AND DISCUSSION

*Selection and preparation of molecular targets* - The molecular targets were retrieved from PDB by considering the lowest values resolution among similar structures and the presence of a ligand in the binding site. As a result, the BraMMT was constructed using 35 molecular targets. Table I shows the PDB code of the selected targets with their respective resolutions. Among them, seven were hydrolases, four isomerases, eight oxidoreductases, eight transferases, four lyases, one cell signaling protein, two transporter proteins, and one cytokine inhibitory protein. In general, all protonation states were kept at pH 7.4. However, 1LF3, 2ANL, 3BPF, and 3FNU targets were present in the digestive vacuole of the parasite; therefore, the protonated state was adjusted to pH 4.0.

The inclusion of water molecules in the binding site was carefully checked by visual inspection. Thus, only water molecules that performed at least two hydrogen bonds between the crystallographic ligand and the respective target remained in the binding site,^10^, such as 1LYX, 1O5X, 1QNG, 1TV5, 1YWG, 2AAW, 2Q8Z, 2VN1, 3CLV, 3FNU, 3N3M, 3PHC, 4C81, 4T64, and 4N0Z. The inclusion of water molecules is a crucial issue since such molecules can affect the ability to predict ligand binding, in which coordination is stabilised by the presence of an explicit water molecule.^7^ ^,^ ^10^

In addition, the TDR targets platform was used to obtain the druggability of major pathogens of tropical diseases, including *Mycobacterium leprae* and *M. tuberculosis*; the protozoa *Leishmania major*, *Trypanosoma brucei*, *T. cruzi*, *P. falciparum*, *P. vivax*, and *Toxoplasma gondii*; and the helminths *Brugia malayi* and *Schistosoma mansoni*.^22^ Using TDR platform, the molecular targets were evaluated according to their druggability to determine the feasibility of the selected targets for SBDD studies, which evaluated the ability of the protein to bind with high affinity and specificity to small compounds.^22^ As a result, Table I shows the druggability values ranging from 0 to 1, which values equal or greater than 0.6 are considered relevant. As seen in Table I, 51.4% (18) of the selected targets (1LF3, 1LYX, 1NHW, 1O5X, 1RL4, 1TV5, 1U4O, 2AAW, 2ANL, 2OK8 2PML, 2Q8Z, 2VFA, 2VN1, 2YOG, 3BPF, 3QS1, 3T64, and 4J56) had druggability values greater than 0.6. In contrast, the targets 3K7Y, 3N3M, 4B1B, 4P7S, 3TLX, PfATP6, and PfHT were not evaluated by TDR because they were not present on the platform.

**TABLE I:** Molecular targets, pH of the intracellular medium, druggability of the targets obtained from the Tropical Disease Research (TDR targets database), enzyme class, and location of the 35 molecular targets

PDB code	Resolution (Å)	pH	Druggability	Enzymatic class	Location
1LF3	1.8	4.0	0.8	Hydrolase	Digestive vacuole
1LYX	1.9	7.4	0.8	Isomerase	Cytoplasm
1NHW	2.3	7.4	0.8	Oxidoreductase	Apicoplast
1O5X	1.1	7.4	0.8	Isomerase	Cytoplasm
1QNG	2.1	7.4	^*^	Isomerase	Cytoplasm
1RL4	2.1	7.4	0.8	Hydrolase	Apicoplast
1TV5	2.4	7.4	1.0	Oxidoreductase	Cytoplasm and Nucleo
1U4O	1.7	7.4	0.8	Oxidoreductase	Cytoplasm
1YWG	2.6	7.4	^*^	Oxidoreductase	Cytoplasm
2AAW	2.4	7.4		Transferase	Cytoplasm
2ANL	3.3	4.0	0.8	Hydrolase	Digestive vacuole
2OK8	2.4	7.4	0.3	Oxidoreductase	Apicoplast
2PML	2.6	7.4	0.9	Transferase	Cytoplasm
2Q8Z	1.8	7.4	0.8	Lyase	Nucleo
2VFA	2.0	7.4	0.8	Transferase	Apicoplast
2VN1	2.3	7.4	0.6	Isomerase	Nucleo
2YOG	1.5	7.4	0.8	Transferase	Nucleo
3AZB	2.6	7.4	^*^	Lyase	Cytoplasm
3BPF	2.9	4.0	0.6	Hydrolase	Digestive vacuole
3CLV	1.8	7.4	0.3	Signaling protein	Cytoplasm
3FNU	3.0	4.0	^*^	Hydrolase	Digestive vacuole
3K7Y	2.8	7.4	^**^	Transferase	Cytoplasm
3N3M	1.4	7.4	^**^	Lyase	Apicoplast
3PHC	2.0	7.4	0.2	Transferase	Nucleo
3QS1	3.1	4.0	0.8	Hydrolase	Digestive vacuole
3T64	1.6	7.4	0.6	Hydrolase	Nucleo
3TLX	2.7	7.4	^**^	Transferase	Cytoplasm and mitochondria
4B1B	2.9	7.4	^**^	Oxidoreductase	Cytoplasm
4C81	1.5	7.4	^*^	Lyase	Apicoplast
4J56	2.3	7.4	0.9	Oxidoreductase	Cytoplasm
4N0Z	1.7	7.4	^*^	Oxidoreductase	Cytoplasm
4P7S	2.8	7.4	^**^	Cytokine Inhibitor	Cytoplasm
4QOX	2.7	7.4	0.5	Transferase	Cytoplasm
PfATP6	3.1+	7.4	^**^	Transporter	Membrane
PfHT	3.1+	7.4	^**^	Transporter	Membrane

PDB: protein data bank; +: resolution of the templates used to build the models - 1IWO and 3O7Q for PfATP6 and PfHT, respectively; *: targets present in the TDR targets database, but without druggability values; **: targets not present in the TDR targets database.

**Fig. 1:**
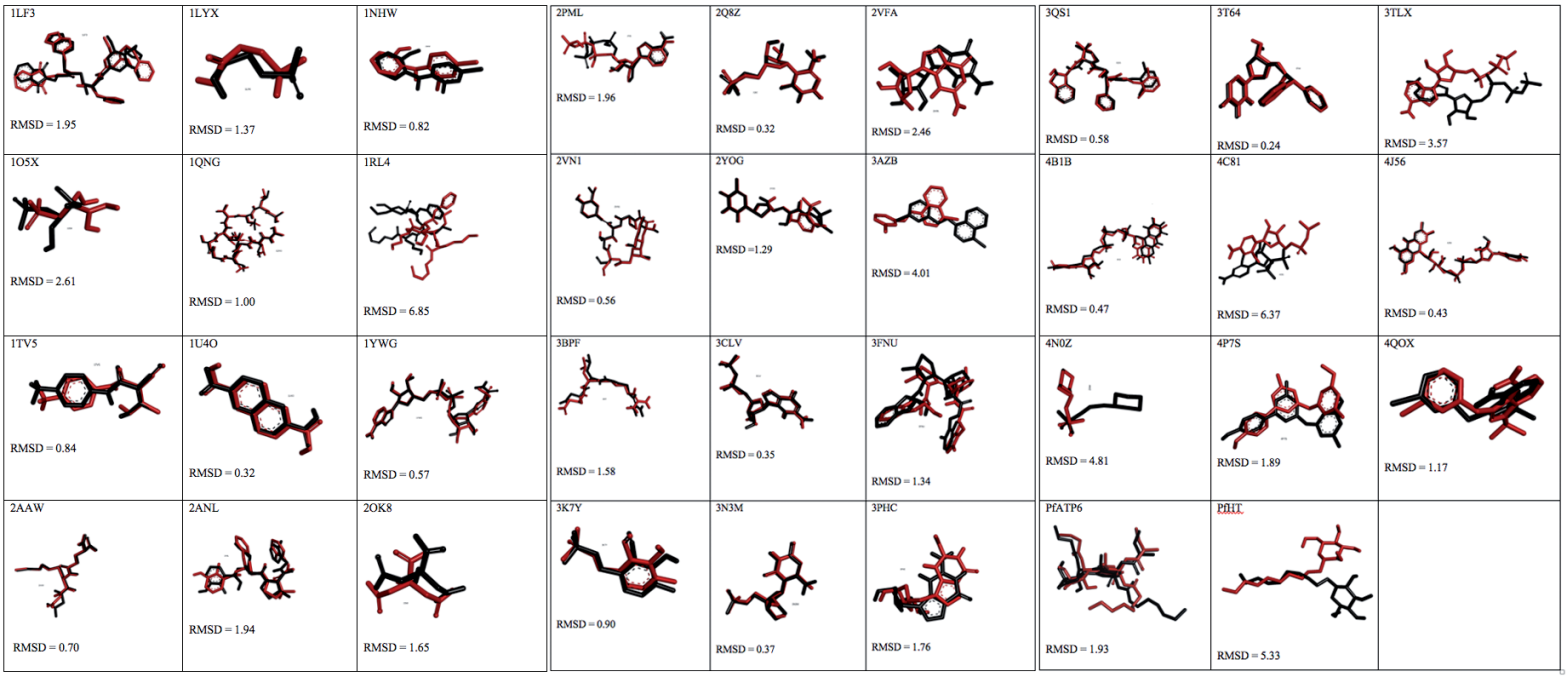
evaluation of AutoDock Vina by molecular redocking, receiver-operator characteristic (ROC) curve, and area under the ROC curve (AUC). Crystallographic and re-docking ligands, in black and red, respectively. Root-mean-square deviation (RMSD) values are in Å.

*Evaluation of molecular docking* - The docking methodology was evaluated by re-docking and the ROC curve.^23^ The re-docking process shows the following: (i) the ability of the methodology to predict the correct positioning of the ligand docked into the binding site. It consists of the structural alignment between the crystallographic ligand and the pose obtained by the docking simulation. This alignment is analysed by RMSD of the heavy atoms positions. The threshold value is 2.0 Å, even though RMSD values of 2.5 Å for ligands with several dihedral angles are considered acceptable^11^; (ii) the affinity between the ligand and receptor (binding energy) expressed by score functions; (iii) the suitability of the configurations parameters, such as size of the grid box and atomic coordinates, for the respective molecular target; and (iv) the main forces for molecular recognition, as aided by another program, such as LigPlot or Discovery Studio. Fig. 1 shows re-docking results among the molecular targets studied. Among the 35 molecular targets, 80% (28) had RMSD values lower than 2.5 Å. In contrast, only 105X, 1RL4, 3AZB, 3TLX, 4C81, 4N0Z, and PfHT molecular targets obtained values that ranged from 2.61 Å to 6.37 Å. In addition, because of the random character of Autodock Vina, there was no significant difference in the binding energy after simulation in triplicate, suggesting that it is robust for virtual screening experiments^11^ [see Supplementary data (Table II)].

**Fig. 2:**
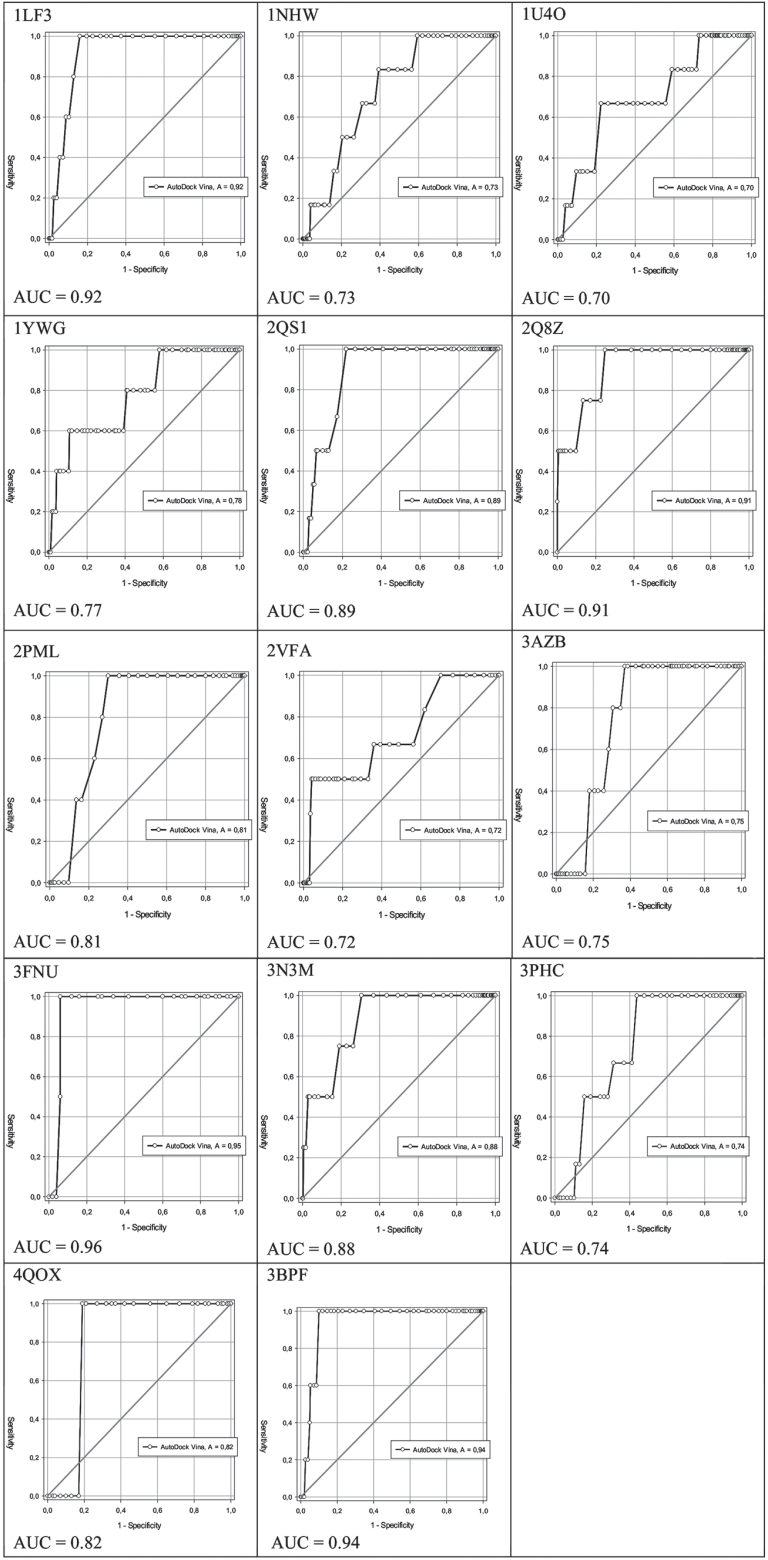
ability of selected targets 1LF3, 1NHW, 1U4O, 1YWG, 2QS1, 2Q8Z, 2PML, 2VFA, 3AZB, 3FNU, 3N3M, 3PHC, 4QOX, and 3BPF to discriminate more than 70% of true positives from false negative.

Furthermore, our results were improved by the ROC curve and AUC calculations. ROC curve and AUC are statistical methodologies to discriminate false-positive and true-positive results from a test. The curve ROC is a graph that uses a binary system classification to discriminate active compounds from inactive. This graph shows specificity on the X axis for true positive compounds (1-specificity) and sensitivity on the Y axis for false positive compounds with value 0.^24^ The quality of a probabilistic classification can be measured by the AUC, which measures how the ROC curve separates two classes with a reference threshold value. AUC values close to 1 indicate classification compounds with 100% accuracy, whereas AUC values less than 0.5 indicate a random process. It has a confidence interval of 95%.^23^ Hence, the ROC curve was constructed for each molecular target, in which the number of active compounds and decoys ranged from two to nine and 50 to 450, respectively [see Supplementary data (Table IV)]. In addition, for the molecular targets 1LYX, 1NHW, 1QNG, 1YWG, 3TLX, and 4C81, it was necessary to select a different organism with the same target. The different targets from other organisms had an identity range of 34-64% [as showed in Supplementary data (Table III)]; therefore, they can use the respective crystallographic ligand found into the binding site.^18^ Fig. 2 shows selected ROC curves, which represents 40% (14) of the total molecular targets. These targets (1LF3, 1NHW, 1U4O, 1YWG, 2QS1, 2Q8Z, 2PML, 2VFA, 3AZB, 3FNU, 3N3M, 3PHC, 4QOX, and 3BPF) could discriminate more than 70% of true positives from false negatives, which have AUC values of 0.92, 0.73, 0.70, 0.77, 0.89, 0.91, 0.81, 0.72, 0.75, 0.96, 0.88, 0.74, 0.82, and 0.94, respectively.

**Fig. 3:**
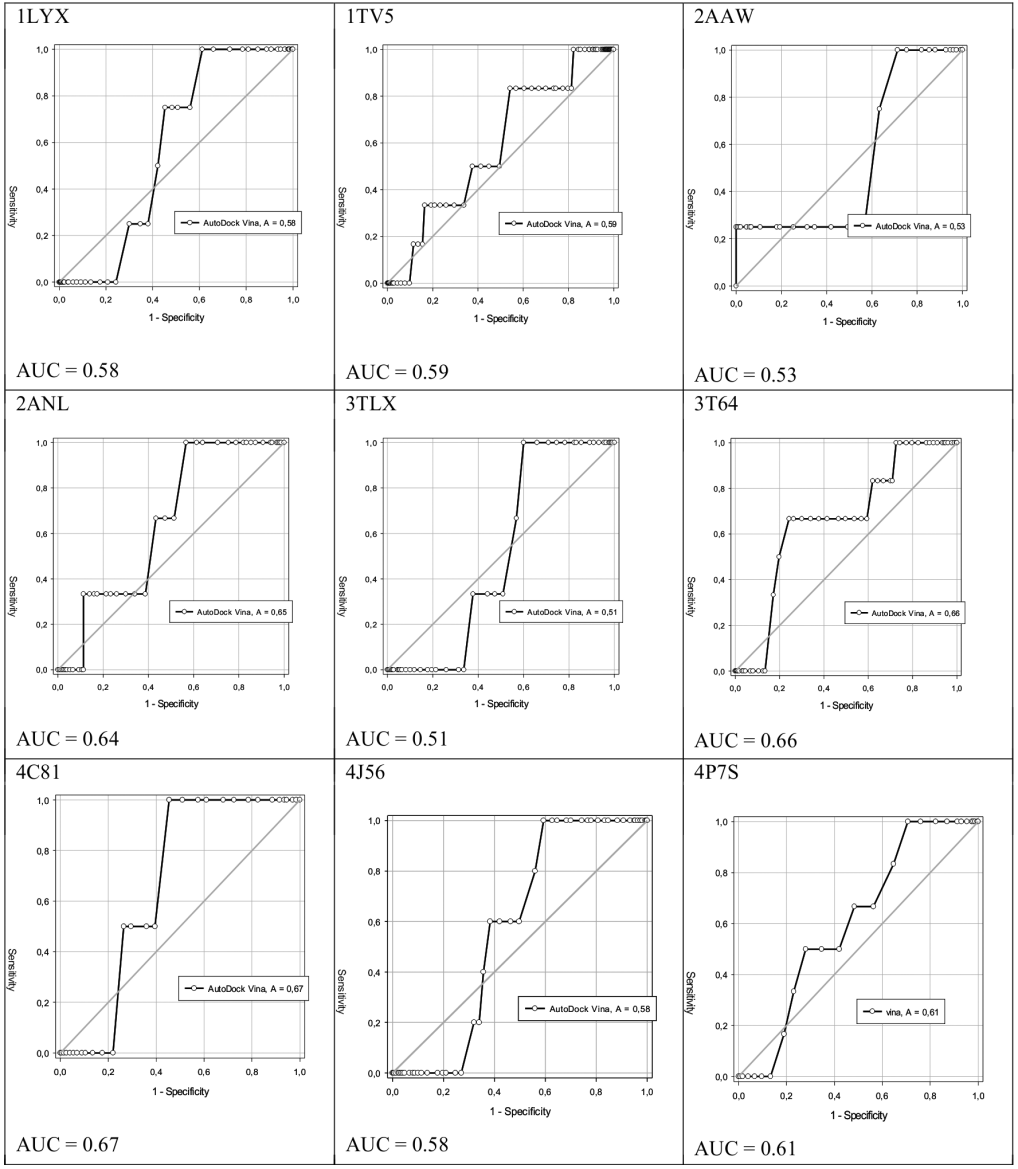
ability of selected targets 1LYX, 1TV5, 2AAW, 2ANL, 3TLX, 3T64, 4C81, 4J56, and 4P7S to discriminate 50-70% of true positives from false negatives.

In contrast, 26% (nine) of the targets 1LYX, 1TV5, 2AAW, 2ANL, 3TLX, 3PHC, 3T64, 4C81, 4J56, and 4P7S had AUC values of 0.58, 0.59, 0.53, 0.64, 0.51, 0.66, 0.67, 0.58, and 0.61, respectively, being able to discriminate true positives from false negatives at 51-67%. In other words, 26% of the targets had AUCs greater than 0.5 and less than 0.7, showing that methodology is not random (Fig. 3). However, these results suggest that another docking parameters or methodology should be used to improve the specificity and selectivity.

**Fig. 4:**
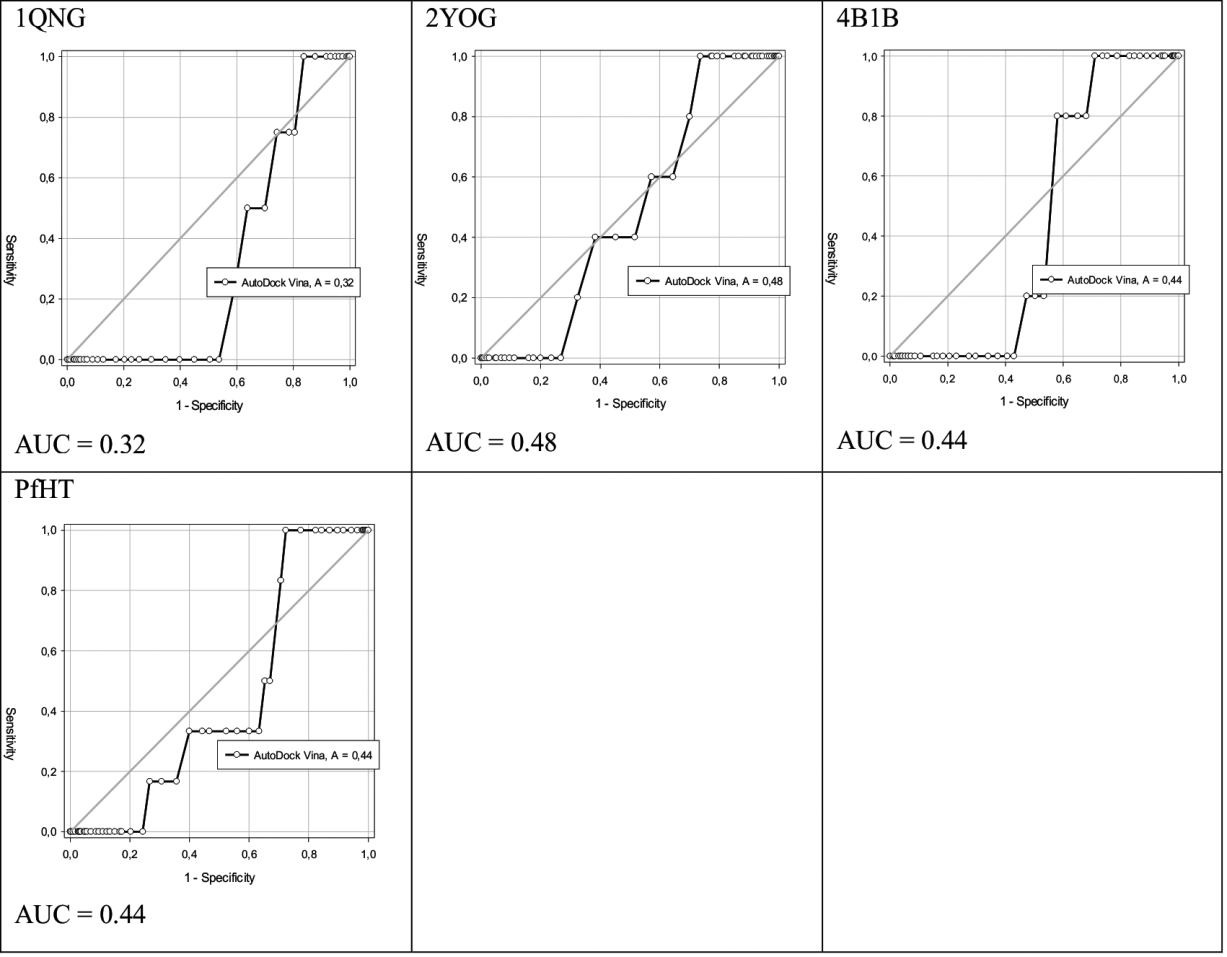
ability of selected targets 1QNG, 2YOG, 4B1B, and PfHT to discriminate less than 50% of true positives from false negatives.

Finally, targets 1QNG, 2YOG, 4B1B, and PfHT (11%) had values of 0.32, 0.48, 0.44, and 0.44, respectively, lower than 0.5 (Fig. 4), showing that the molecular docking methodology could not discriminate true false negatives for this subset of receptors and was considered a random process.

Moreover, for the 1O5X, 1RL4, 2OK8, 2VN1, 3CLV, 3K7Y, 4N0Z, and PfATP6 (~ 22%) targets, it was not possible to perform the ROC curve analysis as they did not have sufficient active compounds. It is noteworthy that the 1O5X, 1RL4, and 4N0Z targets did not have acceptable RMSD values, suggesting less than 0.5.

**Fig. 5:**
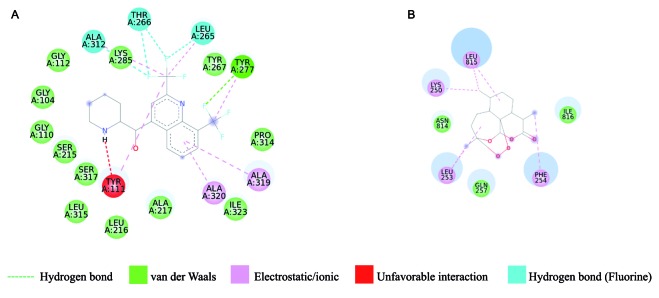
intermolecular interactions between ligands and molecular targets. (A) 1NHW-mefloquine; (B) PfATP6-artemisinin.

*Virtual screening of antimalarial molecules* - The BraMMT data bank was evaluated against antimalarial molecules present on the list of World Health Organization (WHO) drugs. BraMMT, through the OCTOPUS engine, should predict the correct molecular target of these drugs. Owing to the large amount of data, the 3BPF (falcipain-II), 1NHW (*P. falciparum* enoyl-acyl-carrier-protein reductase), and PfATP6 (*P. falciparum* ATPase calcium pump ortholog) molecular targets against the compounds quinine (CHEMBL387326), mefloquine (CHEMBL416956), and artemisinin (CHEMBL269671), respectively, were selected to exemplify the results. Fig. 5 shows 2D ligand-receptor interactions maps. In general, these compounds complex with their receptors through van der Waals interactions and hydrogen bonds. However, they can participate in specific interactions with each receptor. For example, unfavorable interactions may occur between compound mefloquine and 1NHW, but this negative contribution is compensated by others interactions, such as van der Waals, hydrogen bond, and electrostatic interactions of the fluorine atoms of mefloquine with Leu265, Thr266, and Ala312 of 1NHW (Fig. 5A).

Finally, the mechanism of action of artemisinin and its derivatives is still controversial. A possible mechanism is through inhibition of the Ca^2+^ ATPase protein present in the sarcoplastic reticulum, called the *P. falciparum* Atpase orthological calcium pump (PfATP6). Its inhibition can lead to increased concentration of cytoplasmic Ca^2+^, compromising the signaling pathways of the parasite proposed by^25^. Our results confirmed the molecular mechanism of artemisinin (Fig. 5B), suggesting PfATP6 as a possible target of artemisinin.^26^ Therefore, BraMMT is able to select the best molecular target for the compound in this study.

**Fig. 6:**
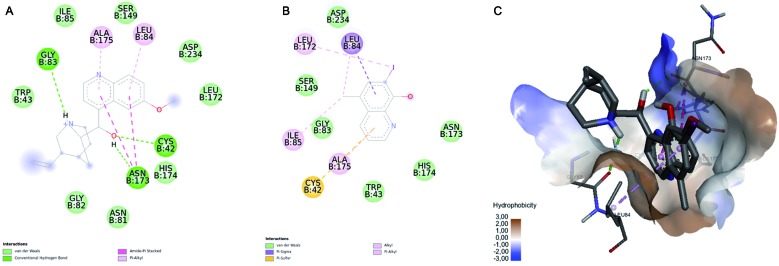
intermolecular interactions between ligands and molecular targets. (A) 3BPF-quinine; (B) 3BPF-clioquinol; (C) Clioquinol (red) and quinine (blue) into the falcipain-II binding site.

Finally, the mechanism of action of quinine is related to the inhibition of heme polymerisation by *P. falciparum*, present in the digestive vacuole. During the intra-erythrocyte cycle, the parasite internalises and degrades the hemoglobin present in the erythrocyte, using amino acids as an energy source. The hemoglobin digestive process is performed using proteolytic enzymes, such as plasmepsins and falcipain, where the parasite has developed a strategy to promote the polymerisation of the heme group to form hemozoin crystals, also known as malaria pigment.^27^ Thus, the hemozoin crystal ceases to be toxic to the parasite; however, it becomes highly allergenic to the vertebrate host and is responsible for the febrile accesses that characterise the disease.^28^ Fig. 6A represents the interactions present between quinine and the falcipain-II molecular target. This finding suggested a new mechanism of action for quinine, namely protease inhibition. Previous reports have shown a similar drug, clioquinol, with protease activity.^29^ ^,^ ^30^ Our results suggested that as these drugs have the same quinolinic ring pharmacophoric moiety, they can share a similar pharmacological mechanism. Clioquinol is able to inhibit methionine aminopeptidases from *M. tuberculosis* ^29^ and active proteasomes.^30^ Our hypothesis was to evaluate the docking of clioquinol against falcipain-II using Autodock Vina (Fig. 6B). The docking binding energy values were -5.5 Kcal/mol and -6.9 Kcal/mol for clioquinol and quinine, respectively. As can be observed by Fig. 6A-B, falcipain-II had better recognition of quinine, resulting in more intermolecular interactions points than clioquinol, explaining a low binding energy. In addition, the overlay of the lowest energy docked pose of each drug showed that the quinolinic rings had similar orientation into the binding site of falcipain-II. In summary, the quinolinic moiety of both drugs could be recognised by Leu84, Ile85, Leu172, Ser149, Asn173, His174, and Ala175 amino acids, suggesting a proteolytic inhibitory mechanism of falcipain-II.

*Evaluation of molecular dynamics* - After to run the molecular dynamics using amber score, a value of -19.41 kcal/mol and -28.07 kcal/mol was obtained for clioquinol and quinine, these results suggest that clioquinol is a potential protease inhibitor, considering that the crystallographic inhibitor presents value of -12.75 kcal/mol.

On the basis of our *in silico* studies, we performed the *in vitro* antiplasmodial activity of clioquinol.

*Antiplasmodial activity* - Clioquinol was considered active and selective against *P. falciparum in vitro*, with an IC_50_ value of 0.56 µM and a high SI of 178.6 (Table II). These findings are in agreement with a recently set of criteria for the development of new antimalarial compounds.^21^

**TABLE II:** *In vitro* antiplasmodial activity, cytotoxicity, and SI of clioquinol

Compound	IC_50_ (µM) ± SD		SI
	*Plasmodium falciparum*	WI-26-VA4		
Clioquinol	0.56 ± 0.03	> 100	178.6
Chloroquine	0.80 ± 0.14	> 100	125.0

SD: standard deviation; SI: selectivity index.

These criteria include: (i) a preliminary knowledge of the structure-activity relationship; (ii) a cutoff *in vitro* potency around 1 μM; and (iii) SI greater than 10-fold against a human cell line.

## CONCLUSIONS

In this work, we carried out 18.150 vHTS simulations, comparable to biological assays, which would have been time consuming and expensive if performed under similar experimental conditions. Thus, the vHTS methodology is a tool to discover the most active molecules inside a bank of millions of compounds and to reduce the number of molecules used in experiments, thus reducing costs by using a rational drug development process.

Furthermore, BraMMT was evaluated by traditional methodology metrics, such as RMSD, ROC curve, and AUC. It was possible to conclude that 77% of the targets had RMSD values lower than 2 Å, proving that these targets could be used in inverse virtual screening through the AutoDock Vina program. Approximately 72% of the targets had AUC values greater than 0.5, showing that they were able to discriminate true positives from false positives. Although some targets did not have validation parameters, they could be used together with other methodologies, such as molecular dynamics, to avoid false positive results.

Moreover, BraMMT was evaluated against known antimalarial compounds, confirming its capacity to predict the correct molecular target. Our experiments suggested new mechanisms for quinine, as a protease inhibitor, which was confirmed by action and structural similarity between the quinolinic ring of quinine and clioquinol drugs. Molecular dynamics showed an amber score value of -19.41 kcal/mol for clioquinol and it was active and selective against *P. falciparum in vitro*.

Finally, BraMMT is available for vHTS experiments through a new version of the OCTOPUS software. The new version of OCTOPUS can build and evaluate molecular targets, as well as perform docking simulation using Autodock Vina and/or Dock 6. Therefore, it can perform vHTS experiments using an unlimited number of ligands against a database, such as BraMMT.

## References

[1] Keiser MJ, Setola V, Irwin JJ, Laggner C, Abbas AI, Hufeisen SJ (2009). Predicting new molecular targets for known drugs. Nature.

[2] Magariños MP, Carmona SJ, Crowther GJ, Ralph SA, Roos DS, Shanmugam D, et at (2012). TDR targets a chemogenomics resource for neglected diseases. Nucleic Acids Res.

[3] Song CM, Lim SJ, Tong JC (2009). Recent advances in computer-aided drug design. Brief Bioinform.

[4] Sliwoski G, Kothiwale S, Meiler J, Lowe EW (2013). Computational methods in drug discovery. Pharmacol Rev.

[5] Baig MH, Ahmad K, Roy S, Ashraf JM, Adil M, Siddiqui MH (2016). Computer aided drug design success and limitations. Curr Pharm Des.

[6] Woods JM, Bethell RC, Coates JAV, Healy N, Hiscox SA, Pearson BA (1993). 4-Guanidino-2,4-dideoxy-2,3-dehydro-N-acetylneuraminic acid is a highly effective inhibitor both of the sialidase (neuraminidase) and of growth of a wide range of influenza A and B viruses in vitro. Antimicrob Agents Chemother.

[7] Sousa SF, Cerqueira NMFSA, Fernandes PA, Ramos MJ (2010). Virtual screening in drug design and development. Comb Chem High Throughput Screen.

[8] Henrich S, Feierberg I, Wang T, Blomberg N, Wade RC (2010). Comparative binding energy analysis for binding affinity and target selectivity prediction. Proteins Struct Funct Bioinforma.

[9] Carregal AP, Comar M, Alves SN, de Siqueira JM, Lima LA, Taranto AG (2012). Inverse virtual screening studies of selected natural compounds from Cerrado. Int J Quantum Chem.

[10] Elokely KM, Doerksen RJ (2013). Docking challenge protein sampling and molecular docking performance. J Chem Inf Model.

[11] Jaghoori MM, Bleijlevens B, Olabarriaga SD (2016). 1001 ways to run AutoDock Vina for virtual screening. J Comput Aided Mol Des.

[12] Maia EHB, Campos VA, Santos BR, Costa MS, Lima IG, Greco SJ (2017). Octopus a platform for the virtual high-throughput screening of a pool of compounds against a set of molecular targets. J Mol Model.

[13] Sabbatani S, Fiorino S, Manfredi R (2010). The emerging of the fifth malaria parasite (Plasmodium knowlesi) a public health concern?. Braz J Infect Dis.

[14] Berman HM, Kleywegt GJ, Nakamura H, Markley JL (2014). The protein data bank archive as an open data resource. J Comput Aided Mol Des.

[15] da Fonseca AL, Nunes RR, Braga VML, Comar M, Alves RJ, Varotti FP (2016). Docking, QM/MM, and molecular dynamics simulations of the hexose transporter from Plasmodium falciparum (PfHT). J Mol Graph Model.

[16] Guimarães DSM, da Fonseca AL, Batista R, Comar M, de Oliveira AB, Taranto AG (2015). Structure-based drug design studies of the interactions of ent-kaurane diterpenes derived from Wedelia paludosa with the Plasmodium falciparum sarco/endoplasmic reticulum Ca2+-ATPase PfATP6. Mem Inst Oswaldo Cruz.

[17] Trott O, Olson AJ (2009). AutoDock Vina improving the speed and accuracy of docking with a new scoring function, efficient optimization, and multithreading. J Comput Chem.

[18] D'Alfonso G.Tramontano A.Lahm A (2001). Structural conservation in single-domain proteins implications for homology modeling. J Struct Biol.

[19] Cheng T, Li Q, Zhou Z, Wang Y, Bryant SH (2012). Structure-based virtual screening for drug discovery a problem-centric review. AAPS J.

[20] Gaulton A, Bellis LJ, Bento AP, Chambers J, Davies M, Hersey A (2012). ChEMBL a large-scale bioactivity database for drug discovery. Nucleic Acids Res.

[21] Katsuno K, Burrows JN, Duncan K, van Huijsduijnen RH, Kaneko T, Kita K (2015). Hit and lead criteria in drug discovery for infectious diseases of the developing world Nat Rev Drug. Discovery.

[22] Agüero F, Al-Lazikani B, Aslett M, Berriman M, Buckner FS, Campbell RK (2008). Genomic-scale prioritization of drug targets the TDR Targets database. Nat Rev Drug Discov.

[23] Flach PA, Wu S. (2005). Repairing concavities in ROC curves.. IJCAI Int Jt Conf Artif Intell..

[24] Kellenberger E, Foata N, Rognan D (2008). Ranking targets in structure-based virtual screening of three-dimensional protein libraries methods and problems. J Chem Inf Model.

[25] Hotta CT, Gazarini ML, Beraldo FH, Varotti FP, Lopes C, Markus RP (2000). Calcium-dependent modulation by melatonin of the circadian rhythm in malarial parasites. Nat Cell Biol.

[26] Valderramos SG, Scanfeld D, Uhlemann A-C, Fidock DA, Krishna S (2010). Investigations into the role of the Plasmodium falciparum SERCA (PfATP6) L263E mutation in artemisinin action and resistance. Antimicrob Agents Chemother.

[27] Sullivan DJ (2002). Theories on malarial pigment formation and quinoline action. Int J Parasitol.

[28] Bray PG, Janneh O, Raynes KJ, Mungthin M, Ginsburg H, Ward SA (2000). Cellular uptake of chloroquine is dependent on binding to ferriprotoporphyrin IX and is independent of NHE activity in Plasmodium falciparum. J Cell Biol.

[29] Olaleye O, Raghunand TR, Bhat S, Chong C, Gu P, Zhou J (2011). Characterization of clioquinol and analogues as novel inhibitors of methionine aminopeptidases from Mycobacterium tuberculosis.. Tuberculosis.

[30] Chen D, Cui QC, Yang H, Barrea RA, Sarkar FH, Sheng S (2007). Clioquinol, a therapeutic agent for Alzheimer's disease, has proteasome-inhibitory, androgen receptor-suppressing, apoptosis-inducing, and antitumor activities in human prostate cancer cells and xenografts. Cancer Res.

