# Supplementary material for: Brazilian malaria molecular targets (BraMMT): selected receptors for virtual high-throughput screening experiments
Source: Mem Inst Oswaldo Cruz. 2019 Feb 25;114:e180465. doi: 10.1590/0074-02760180465 (PMC6388387; doi:10.1590/0074-02760180465)
Supplement: Supplementary file 1 [file 1678-8060-mioc-114-e180465-s.pdf]

TABLE I

Protein data bank (PDB) code of the selected targets with the code of their respective crystallographic binders X, Y and Z coordinates and just box size with the linker for the molecular targets

		GRID BOX
Targets	Ligand code	Number of dimension points
1LF3	EH5	Size_x = 16.215<br>Size_y = 6.85<br>Size_z = 27.605
1LYX	PGA	Size_x = 21.876<br>Size_y = -2.117<br>Size_z = 7.919
1NHW	TCC	Size_x = 52.704<br>Size_y = 93.452<br>Size_z = 34.625
1O5X	3PY	Size_x = 20.282<br>Size_y = -19.183<br>Size_z = 22.197
1QNG	PRD_000142	Size_x = 10<br>Size_y = 18<br>Size_z = 16
1RL4	BL5	Size_x = -13.342<br>Size_y = 32.081<br>Size_z = -12.449
1TV5	A26	Size_x = 38.204<br>Size_y = 35.063<br>Size_z = 36.245
1U4O	NDD	Size_x = 10<br>Size_y = 6<br>Size_z = 4
1YWG	NAD	Size_x = 72.893<br>Size_y = 26.064<br>Size_z = 10.926
2AAW	GTX	Size_x = 1.32<br>Size_y = 41.126<br>Size_z = 37.762
2ANL	PRD_000566	Size_x = 54.924<br>Size_y = 13.448<br>Center_z = 25.686
2OK8	FLC	Size_x =- 82.404<br>Size_y = 26.218<br>Size_z = -7.871
2PML	ANP	Size_x = -9.653

CC BY

		Size_y = 9.26<br>Size_z = -20.059
2Q8Z	NUP	Size_x = 8.087<br>Size_y = -27.159<br>Size_z = -23.912
2VFA	5GP	Size_x = -32.364<br>Size_y = -61.727<br>Size_z = -31.813
2VN1	FK5	Size_x = 0.208<br>Size_y = 33.661<br>Size_z = 16.072
2YOG	74X	Size_x = 15.189<br>Size_y = 9.23<br>Size_z = 1.839
3AZB	KM1	Size_x = 20.479<br>Size_y = -23.149<br>Size_z = 9.023
3BPF	E64	Size_x = -36.87<br>Size_y = 31.066<br>Size_z = -47.069
3CLV	GDP	Size_x = -41.436<br>Size_y = -4.483<br>Size_z = -10.534
3FNU	006	Size_x = -8.213<br>Size_y = -49.117<br>Size_z = -77.022
3K7Y	PLP	Size_x = 21.365<br>Size_y = 30.311<br>Size_z = 26.325
3N3M	NUP	Size_x = 8<br>Size_ y = 2<br>Size_z = 8
3PHC	IM5	Size_x = -16.593<br>Size_y = 9.689<br>Size_z = -18.996
3QS1	006	Size_x = 27.799<br>Size_y = -10.291<br>Size_z = 4.252
3TLX	ATP	Size_x = 24.506<br>Size_y = -25.17<br>Size_z = 55.808
3T64	DU3	Size_x = 10.089<br>Size_y = 46.06

		Size_z = -1.47
4B1B	FAD	Size_x = -26.906<br>Size_y = 5.767<br>Size_z = -24.052
4C81	CDP	Size_x = -32.194<br>Size_y = -35.597<br>Size_z = 19.38
4N0Z	MPO	Size_x = 20.377<br>Size_y = 0.64<br>Size_z = -1.164
4P7S	2OK	Size_x = -30.409<br>Size_y = -23.725<br>Size_z = -3.746
4QOX	DXR	Size_x = 29.978<br>Size_y = -13.348<br>Size_z = -0.466
4J56	FAD	Size_x = -31.168<br>Size_y = -108.197<br>Size_z = 197.847
PfATP6	Thapsigargin	Size_x = -5.142<br>Size_y = -48.212<br>Size_z = 8.979
PfHT	B-nonylglucoside	Size_x = 84.939<br>Size_y = 54.482<br>Size_z = -35.193

TABLE II

Root mean square deviation (RMSD) values for re-docking. Binding energy between ligand and receptor (Kcal / mol) in triplicate

PDB Code	RMSD	1º	2º	3º	Average
3N3M	0.38	-9.5	-9.6	-9.6	-9.6
1U4O	0.32	-8.1	-8.1	-8.1	-8.1
1QNG	1.00	-7.8	-7.8	-7.8	-7.7
4P7S	1.90	-6.0	-6.1	-6.1	-6.0
2YOG	1.30	-8.4	-8.5	-8.5	-8.4
4J56	0.44	-13.0	-13.0	-13.0	-13.0
2PML	1.96	-6.9	-6.8	-7.0	-6.9
3T64	0.25	-8.3	-8.3	-8.3	-8.1
3AZB	4.01	-6.3	-6.0	-6.3	-6.3
1NHW	0.82	-8.3	-8.3	-8.3	-8.3
3QS1	0.59	-10.4	-10.4	-10.4	-10.4
1LF3	1.96	-9.6	-9.7	-9.6	-9.6
2ANL	1.94	-9.3	-9.3	-9.3	-9.3
1TV5	0.84	-9.3	-9.3	-9.3	-9.3
2Q8Z	0.32	-9.7	-9.7	-9.7	-9.7
1LYX	1.37	-5.6	-5.6	-5.5	-5.6
3BPF	1.58	-6.3	-6.3	-6.3	-6.3
4N0Z	4.82	-4.3	-4.3	-4.2	-4.3
4B1B	0.47	-12.3	-12.2	-12.2	-12.3
1O5X	2.61	-5.3	-5.5	-5.5	-5.3
1RL4	6.85	0.0	0.0	0.0	0.0
3FNU	1.35	-9.2	-9.0	-9.3	-9.2
4QOX	1.17	-8.9	-8.9	-8.9	-8.9
2VFA	2.47	-5.8	-5.8	-5.8	-5.8
2VN1	0.57	-14.6	-14.6	-14.6	-14.6
4C81	6.37	-6.0	-5.9	-6.2	-6.0
3TLX	3.57	-5.9	-5.9	-5.9	-5.9
3PHC	1.77	-8.3	-8.3	-8.3	-8.3
3CLV	0.36	-11.7	-11.7	-11.7	-11.7
1YWG	0.58	-10.7	-10.5	-10.6	-10.7
2AAW	0.71	-9.1	-8.8	-9.1	-9.1
2OK8	1.66	-2.0	-2.0	-2.0	-2.0
3K7Y	0.91	-7.7	-7.7	-7.6	-7.7
PfATP6	1.93	-7.2	-6.9	-7.7	-7.2
PfHT	5.33	-5.7	-5.7	-5.6	-5.7

PDB: protein data bank.

TABLE III

Quantity of assets and decoys used to perform the receiver-operator characteristic (ROC) curve and area under the ROC curve (AUC). With their respective organisms used and the degree of identity found after performing the alignment in the BLAST program

PDB code	Active	Decoys	Organism	Identity (%)
1LF3	5	250	*Plasmodium falciparum*	-
1LYX	4	400	*Homo sapiens*	*43*
1NHW	6	450	*Toxoplasma gondii*	51
1QNG	4	400	*Homo sapiens*	*61*
1TV5	6	450	*P. falciparum*	-
1U4O	6	300	*P. falciparum*	-
1YWG	6	250	*H. sapiens*	*64*
2AAW	3	150	*P. falciparum*	-
2ANL	3	150	*P. falciparum*	-
2PML	6	300	*P. falciparum*	-
2Q8Z	4	350	*P. falciparum*	-
2VFA	6	450	*H. sapiens*	-
2YOG	5	250	*P. falciparum*	-
3AZB	6	300	*P. falciparum*	-
3BPF	6	300	*P. falciparum*	-
3FNU	2	50	*P. falciparum*	-
3N3M	4	350	*P. falciparum*	-
3PHC	6	350	*P. falciparum*	-
3QS1	6	300	*P. falciparum*	-
3TLX	4	220	*H. sapiens*	*52*
3T64	6	350	*P. falciparum*	-
4B1B	6	300	*P. falciparum*	-
4C81	2	200	*Escherichia coli*	34
4J56	6	300	*P. falciparum*	-
4P7S	6	300	*P. falciparum*	-
4QOX	2	100	*P. falciparum*	-
PfHT	6	300	*P. falciparum*	-

PDB: protein data bank.

cc BY

TABLE IV

Inhibition constant values (Ki) and IC50 of the targets present in the BRAMMT obtained in the ChemBL Platform

PDB code: 1U4O - ID ChemBL: CHEMBL6071

IC_{50} (nM)	MM	Compound ID
IC_{50} = 92500	89.05	CHEMBL15976
IC_{50} = 2640	518.55	CHEMBL51483
IC_{50} = 87300	429.33	CHEMBL245025
IC_{50} = 191000	443.36	CHEMBL390487
IC_{50} = 3130	532.5	CHEMBL242019
IC_{50} = 8250	557.51	CHEMBL390488

PDB code: 2YOG - ID ChemBL: CHEMBL2176852

Ki (nM)	MM	Compound ID
Ki = 90000	410.88	CHEMBL442081
Ki = 478.87	310.00	CHEMBL392137
Ki = 88000	462.81	CHEMBL391451
Ki = 25000	478.87	CHEMBL1928432
Ki = 132000	390.39	CHEMBL2178477

Código 3N3M - ID ChemBL: CHEMBL5173

Ki (nM)	MM	Compound ID
Ki = 210000	324.18	CHEMBL214393
Ki = 1000	325.17	CHEMBL463480
Ki = 910000000	349.19	CHEMBL1164065
Ki = 22100	339.2	CHEMBL2178721

PDB code: 2ANL - ID ChemBL: CHEMBL1741262

Ki (nM)	MM	Compound ID
Ki = 5000	552.62	CHEMBL177718
Ki = 478	796.54	CHEMBL410049
Ki = 35	760.51	CHEMBL373047

PDB code: 4J56 - ID ChemBL: CHEMBL4547

IC_{50} (nM)	MM	Compound ID
IC50=8000	270.55	CHEMBL144530
IC50 = 10000	181.17	CHEMBL206713
IC50 = 10000	226.17	CHEMBL206140
IC50 = 4000	175.14	CHEMBL380630
IC50 = 2000	220.14	CHEMBL380953
IC50 = 30000	236.1	CHEMBL380842

PDB code: 4P7S - ID ChemBL: CHEMBL2176864

CC BY

Ki (nM)	MM	Compound ID
Ki = 99000	328.34	CHEMBL175434
Ki = 8600	326.27	CHEMBL2178826
Ki = 15500	342.73	CHEMBL2178828
Ki = 28600	324.3	CHEMBL2178829
Ki = 19300	278.31	CHEMBL2178830
Ki = 122300	334.33	CHEMBL2178831

PDB code: 2PML - ID ChemBL: CHEMBL6169

IC_{50} (nM)	MM	Compound ID
IC_{50} = 11587.77	331.37	CHEMBL506400
IC_{50} = 7900	302.33	CHEMBL524106
IC_{50} = 810	326.35	CHEMBL450347
IC_{50} = 1260	340.38	CHEMBL522222
IC_{50} = 749.89	319.36	CHEMBL496114
IC_{50} = 580	289.33	CHEMBL522223

PDB code: 2Q8Z - ID ChemBL CHEMBL5173

Ki (nM)	MM	Compound ID
Ki = 210000	324.18	CHEMBL214393
Ki = 1000	325.17	CHEMBL463480
Ki = 910000000	349.19	CHEMBL1164065
Ki = 22100	339.2	CHEMBL2178721

PDB code: 3T64 - ID ChemBL: CHEMBL4222

Ki (nM)	MM	Compound ID
Ki = 98000	290.27	CHEMBL11415
Ki = 298000	210.19	CHEMBL89637
Ki = 200	469.53	CHEMBL372524
Ki = 26000	459.39	CHEMBL194269
Ki = 324000	405.34	CHEMBL194650
Ki = 426000	389.45	CHEMBL194473

PDB code: 3AZB - ChemBL: CHEMBL4512

IC_{50} (nM)	MM	Compound ID
IC_{50} = 7000	270.24	CHEMBL44
IC_{50} = 30000	254.24	CHEMBL8145
IC_{50} = 1500	302.24	CHEMBL50
IC_{50} = 20000	238.24	CHEMBL276915
IC_{50} = 8000	302.24	CHEMBL28626

IC_{50} = 2000	286.24	CHEMBL151

PDB code: 3QS1 - ID ChemBL: CHEMBL4687

Ki (nM)	MM	Compound ID
Ki = 16	596.72	CHEMBL113208
Ki = 3.5	705.64	CHEMBL112598
Ki = 3.9	611.53	CHEMBL112468
Ki = 4.1	603.55	CHEMBL443541
Ki = 8.9	640.57	CHEMBL115297
Ki = 53	636.54	CHEMBL116282

PDB code: 2AAW - ID ChemBL: CHEMBL1697656

IC50 (nM)	MM	Compound ID
IC_{50} = 74400	302.19	CHEMBL6246
IC_{50} = 29300	318.19	CHEMBL1688543
IC_{50} = 7000	334.19	CHEMBL1688544

PDB code: 1LF3 - ID ChemBL: CHEMBL4414

Ki (nM)	MM	ID Compostos
Ki = 9	695.84	CHEMBL284955
Ki = 4.3	594.14	CHEMBL33989
Ki = 2	608.17	CHEMBL285177
Ki = 100	799.86	CHEMBL32997
Ki = 4.8	751.86	CHEMBL284534

PDB code: 1TV5 - ID ChemBL: CHEMBL3486

Ki (nM)	MM	Compound ID
Ki = 22000	270.21	CHEMBL973
Ki = 11000	296.24	CHEMBL141732
Ki = 26000	304.34	CHEMBL142996
Ki = 4900	331.36	CHEMBL199572
Ki = 17700	317.34	CHEMBL370865
Ki = 10800	384.22	CHEMBL372561

PDB code: 3BPF - ID ChemBL: CHEMBL5800

Ki (nM)	MM	Compound ID
Ki = 4000	644.98	CHEMBL378223
Ki = 290	357.41	CHEMBL374508
Ki = 120	755.16	CHEMBL271992
Ki = 110	677.09	CHEMBL270278
Ki = 140	725.13	CHEMBL408071

Ki = 370	663.06	CHEMBL407354

PDB code: 4B1B - ID ChemBL: CHEMBL4547

IC_{50} (nM)	MM	Compound ID
IC_{50} = 8000	270.55	CHEMBL144530
IC_{50} = 10000	181.17	CHEMBL206713
IC_{50} = 10000	226.17	CHEMBL206140
IC_{50} = 40000	175.14	CHEMBL380630
IC_{50} = 2000	220.14	CHEMBL380953
IC_{50} = 30000	236.1	CHEMBL380842

Codígo PDB: 3FNU HAP - ID ChemBL: CHEMBL6075

IC_{50} (nM)	MM	ID do compostos
IC_{50} = 690	631.78	CHEMBL231522

PDB code: 4QOX - ID ChemBL: CHEMBL2169725

IC_{50} (nM)	MM	Compound ID
IC_{50} = 4	402.49	CHEMBL2030554
IC_{50} = 177	317.39	CHEMBL1231371

PDB code: 2VFA - ID ChemBL: CHEMBL2360

Ki (nM)	MM	Compound ID
Ki = 500	301.24	CHEMBL267803
Ki = 21000	287.21	CHEMBL19784
Ki = 31000	289.19	CHEMBL20283
Ki = 48100	425.24	CHEMBL83231
Ki = 16000	354.23	CHEMBL88057
Ki = 6700	273.29	CHEMBL82858

PDB code: 4C81 - ID ChemBL: CHEMBL3217375

IC_{50} (nM)	MM	Compound ID
IC_{50} = 768000	403.18	CHEMBL425252
IC_{50} = 797300	401.2	CHEMBL3220831

PDB code: 3PHC - ID ChemBL: CHEMBL5648

Ki (nM)	MM	Compound ID
Ki = 59000	223.27	CHEMBL1275700
Ki = 27000	209.25	CHEMBL1275699
Ki = 191	234.25	CHEMBL269866
Ki = 580	264.28	CHEMBL271387
Ki = 23.3	282.27	CHEMBL498940
Ki = 2.7	282.32	CHEMBL1275659

PDB code: 1LYX - ID ChemBL: CHEMBL4880

Ki (nM)	MM	Compound ID
Ki = 4000	171.05	CHEMBL371668
Ki = 111000	170.06	CHEMBL195520
Ki = 160000	169.07	CHEMBL196442
Ki = 15000	682.89	CHEMBL3142429

PDB code: 1NHW - ID ChemBL: CHEMBL1250368

IC_{50} (nM)	MM	Compound ID
IC_{50} = 15	289.54	CHEMBL849
IC_{50} = 130	265.65	CHEMBL1240784
IC_{50} = 2800	221.64	CHEMBL1240915
IC_{50} = 3000	373.66	CHEMBL1243023
IC_{50} = 490	296.75	CHEMBL1243024
IC_{50} = 2000	364.22	CHEMBL1243120

PDB code: 3TLX - ID ChemBL: CHEMBL4938

Ki (nM)	MM	Compound ID
Ki = 4700000	726.46	CHEMBL607736
Ki = 6700000	663.41	CHEMBL608643
Ki = 6800000	924.72	CHEMBL610134
Ki = 7300000	621.37	CHEMBL609238

PDB code: 1YWG - ID ChemBL: CHEMBL2284

IC_{50} (nM)	MM	Compound ID
IC_{50} = 691830.97	146.14	CHEMBL6466
IC_{50} = 35000	267.24	CHEMBL477
IC_{50} = 208929.61	244.29	CHEMBL52229
IC_{50} = 75000	579.43	CHEMBL294813
IC_{50} = 7000	570.6	CHEMBL303934
IC_{50} = 5000	584.62	CHEMBL63338

PDB code: 1QNG - ID ChemBL: CHEMBL1949

IC_{50} (nM)	MM	Compound ID
IC_{50} = 325000	737.89	CHEMBL151385
IC_{50} = 165000	647.77	CHEMBL434694
IC_{50} = 2600000	548.64	CHEMBL148546
IC_{50} = 260000	681.83	CHEMBL149224

PDB code: PFHT - ID ChemBL: CHEMBL4697

Ki (nM)	MM	Compound ID

CC BY

Ki = 130000	596.38	CHEMBL382864
Ki = 140000	604.81	CHEMBL203809
Ki = 36000	346.46	CHEMBL204956
Ki = 37000	388.54	CHEMBL203759
Ki = 590000	348.43	CHEMBL381073
Ki = 81000	382.49	CHEMBL202600

PDB: protein data bank.

TABLE V

Virtual screening of antimalarial

Targets	1LF3	Δ	1LYX	Δ	1NHW	Δ	1QNG	Δ	1RL4	Δ
Crystal. Ligand.	-9.6		-5.6		-8.3		-7.7		-8.0	
CHEMBL1107	-8.0	-1.6	-5.8	0.2	-8.3	0.0	-7.2	-0.5	-6.0	-2.0
CHEMBL1327821	-8.5	-1.1	-6.7	1.1	-9.6	1.3	-7.9	0.2	-7.6	-0.4
CHEMBL1377	-7.1	-2.5	-5.7	0.1	-7.3	-1.0	-6.6	-1.1	-6.0	-2.0
CHEMBL1450	-8.8	-0.8	-6.4	0.8	-10.0	1.7	-6.8	-0.9	-8.1	0.1
CHEMBL1525826	-6.9	-2.7	-5.6	0.0	-7.2	-1.1	-6.6	-1.1	-6.0	-2.0
CHEMBL1535	-6.1	-3.5	-5.4	-0.2	-7.0	-1.3	-6.5	-1.2	-5.5	-2.5
CHEMBL154166	-7.0	-2.6	-6.3	0.7	-8.8	0.5	-6.4	-1.3	-6.4	-1.6
CHEMBL1999063	-7.7	-1.9	-6.5	0.9	-8.3	0.0	-7.3	-0.4	-7.5	-0.5
CHEMBL2104009	-6.5	-3.1	-5.3	-0.3	-7.8	-0.5	-5.9	-1.8	-5.5	-2.5
CHEMBL2110945	-6.8	-2.8	-6.2	0.6	-8.2	-0.1	-5.8	-1.9	-5.5	-2.5
CHEMBL258608	-8.2	-1.4	-6.7	1.1	-9.6	1.3	-6.9	-0.8	-6.9	-1.1
CHEMBL269671	-7.4	-2.2	-5.7	0.1	-8.7	0.4	-7.3	-0.4	-7.2	-0.8
CHEMBL301267	-6.1	-3.5	-6.6	1.0	-8.6	0.3	-5.9	-1.8	-6.7	-1.3
CHEMBL303933	-8.8	-0.8	-7.2	1.6	-10.3	2.0	-7.9	0.2	-7.1	-0.9
CHEMBL307261	-7.5	-2.1	-5.9	0.3	-8.9	0.6	-7.1	-0.6	-7.0	-1.0
CHEMBL339049	-9.1	-0.5	-7.3	1.7	-9.8	1.5	-8.1	0.4	-7.9	-0.1
CHEMBL35228	-9.3	-0.3	-6.2	0.6	-10.1	1.8	-7.9	0.2	-6.8	-1.2
CHEMBL36	-6.2	-3.4	-5.0	-0.6	-7.0	-1.3	-5.9	-1.8	-6.7	-1.3
CHEMBL380230	-9.7	0.1	-7.1	1.5	-9.8	1.5	-6.8	-0.9	-8.5	0.5
CHEMBL387326	-8.0	-1.6	-5.9	0.3	-8.8	0.5	-7.5	-0.2	-6.8	-1.2
CHEMBL416956	-8.5	-1.7	-5.5	-1.0	-9.0	0.7	-6.9	-0.6	-7.1	-1.9
CHEMBL422330	-7.9	-1.7	-4.6	-1.0	-8.8	0.5	-7.1	-0.6	-6.1	-1.9
CHEMBL506	-6.0	-3.6	-5.5	-0.1	-6.3	-2.0	-6.1	-1.6	-5.4	-2.6
CHEMBL566534	-7.4	-2.2	-5.5	-0.1	-8.5	0.2	-6.6	-1.1	-6.6	-1.4
CHEMBL682	-7.2	-2.4	-6.1	0.5	-8.0	-0.3	-6.8	-0.9	-6.5	-1.5
CHEMBL747	-6.3	-3.3	-5.0	-0.6	-7.6	-0.7	-5.3	-2.4	-6.8	-1.2
CHEMBL76	-6.2	-3.4	-5.4	-0.2	-7.2	-1.1	-6.1	-1.6	-5.6	-2.4

Targets	1TV5	Δ	1U4O	Δ	1YWG	Δ	2AAW	Δ	2ANL	Δ
Crystal. Ligand..	-9.3		-8.1		-10.7		-9.1		-9.3	
CHEMBL1107	-4.5	-4.8	-6.7	-1.4	-6.1	-4.6	-6.6	-2.5	-7.9	-1.4
CHEMBL1327821	-6.1	-3.2	-7.8	-0.3	-9.0	-1.7	-7.5	-1.6	-9.0	-0.3
CHEMBL1377	-8.4	-0.9	-5.9	-2.2	-6.8	-3.9	-5.5	-3.6	-6.9	-2.4
CHEMBL1450	-8.0	-1.3	-8.7	0.6	-7.5	-3.2	-7.6	-1.5	-9.4	0.1
CHEMBL1525826	-9.0	-0.3	-6.0	-2.1	-6.0	-4.7	-5.7	-3.4	-6.3	-3.0
CHEMBL1535	-7.4	-1.9	-5.6	-2.5	-6.1	-4.6	-5.3	-3.8	-6.3	-3.0
CHEMBL154166	-8.6	-0.7	-6.9	-1.2	-6.6	-4.1	-7.0	-2.1	-7.3	-2.0
CHEMBL1999063	-10.1	0.8	-6.3	-1.8	-7.0	-3.7	-5.9	-3.2	-8.0	-1.3
CHEMBL2104009	-7.5	-1.8	-6.4	-1.7	-5.8	-4.9	-5.8	-3.3	-6.4	-2.9

CC BY

CHEMBL2110945	-7.0	-2.3	-6.0	-2.1	-6.5	-4.2	-5.9	-3.2	-6.7	-2.6
CHEMBL258608	-3.9	-5.4	-7.7	-0.4	-7.3	-3.4	-7.2	-1.9	-7.8	-1.5
CHEMBL269671	-7.0	-2.3	-7.4	-0.7	-6.5	-4.2	-6.6	-2.5	-7.9	-1.4
CHEMBL301267	-3.1	-6.2	-7.1	-1.0	-6.0	-4.7	-6.7	-2.4	-7.6	-1.7
CHEMBL303933	-7.2	-2.1	-8.0	-0.1	-9.4	-1.3	-7.7	-1.4	-9.1	-0.2
CHEMBL307261	-6.2	-3.1	-7.7	-0.4	-6.5	-4.2	-6.9	-2.2	-7.8	-1.5
CHEMBL339049	-4.5	-4.8	-7.6	-0.5	-8.6	-2.1	-8.0	-1.1	-9.1	-0.2
CHEMBL35228	-1.5	-7.8	-8.0	-0.1	-8.2	-2.5	-8.0	-1.1	-8.7	-0.6
CHEMBL36	-7.9	-1.4	-6.1	-2.0	-6.2	-4.5	-5.8	-3.3	-6.6	-2.7
CHEMBL380230	-5.3	-4.0	-7.9	-0.2	-7.7	-3.0	-7.3	-1.8	-8.9	-0.4
CHEMBL387326	-8.2	-1.1	-6.9	-1.2	-6.8	-3.9	-6.3	-2.8	-7.7	-1.6
CHEMBL416956	-9.8	-7.7	-7.6	-1.3	-7.8	-3.2	-6.7	-2.2	-7.9	-1.7
CHEMBL422330	-1.6	-7.7	-6.8	-1.3	-7.5	-3.2	-6.9	-2.2	-7.6	-1.7
CHEMBL506	-8.0	-1.3	-5.4	-2.7	-6.6	-4.1	-5.8	-3.3	-6.3	-3.0
CHEMBL566534	-6.9	-2.4	-7.0	-1.1	-6.9	-3.8	-6.3	-2.8	-7.7	-1.6
CHEMBL682	-6.7	-2.6	-6.9	-1.2	-7.8	-2.9	-6.6	-2.5	-7.2	-2.1
CHEMBL747	-7.9	-1.4	-6.1	-2.0	-5.8	-4.9	-5.6	-3.5	-6.7	-2.6
CHEMBL76	-7.7	-1.6	-5.6	-2.5	-6.1	-4.6	-5.0	-4.1	-6.4	-2.9

Targets	2PML	Δ	2Q8Z	Δ	2VN1	Δ	2YOG	Δ	3BPF	Δ
Crystal. Ligand.	-6.9		-9.7		-14.6		-8.4		-6.3	
CHEMBL1107	-8.8	1.9	-5.2	-4.5	-7.9	-6.7	-8.1	-0.3	-6.2	-0.1
CHEMBL1327821	-9.7	2.8	-5.7	-4.0	-9.8	-4.8	-9.8	1.4	-7.1	0.8
CHEMBL1377	-7.3	0.4	-6.8	-2.9	-6.9	-7.7	-7.8	-0.6	-6.2	-0.1
CHEMBL1450	-10.5	3.6	-4.0	-5.7	-9.3	-5.3	-10.3	1.9	-7.5	1.2
CHEMBL1525826	-6.9	0.0	-7.9	-1.8	-6.7	-7.9	-7.7	-0.7	-6.1	-0.2
CHEMBL1535	-7.3	0.4	-6.4	-3.3	-6.9	-7.7	-7.1	-1.3	-6.0	-0.3
CHEMBL154166	-8.2	1.3	-6.3	-3.4	-7.8	-6.8	-8.1	-0.3	-6.5	0.2
CHEMBL1999063	-8.4	1.5	-5.4	-4.3	-8.9	-5.7	-8.5	0.1	-5.6	-0.7
CHEMBL2104009	-7.6	0.7	-4.7	-5.0	-6.8	-7.8	-7.7	-0.7	-5.7	-0.6
CHEMBL2110945	-7.3	0.4	-6.1	-3.6	-7.7	-6.9	-7.7	-0.7	-6.4	0.1
CHEMBL258608	-8.6	1.7	-6.0	-3.7	-8.8	-5.8	-8.1	-0.3	-7.1	0.8
CHEMBL269671	-8.4	1.5	-6.2	-3.5	-8.4	-6.2	-7.1	-1.3	-6.9	0.6
CHEMBL301267	-6.1	-0.8	-5.9	-3.8	-7.8	-6.8	-7.4	-1.0	-6.3	0.0
CHEMBL303933	-9.7	2.8	-5.2	-4.5	-9.8	-4.8	-10.2	1.8	-8.1	1.8
CHEMBL307261	-8.5	1.6	-5.9	-3.8	-8.3	-6.3	-6.8	-1.6	-6.9	0.6
CHEMBL339049	-9.8	2.9	-4.9	-4.8	-10.2	-4.4	-9.4	1.0	-7.8	1.5
CHEMBL35228	-10.0	3.1	-4.9	-4.8	-10.6	-4.0	-9.6	1.2	-7.4	1.1
CHEMBL36	-7.4	0.5	-7.0	-2.7	-6.6	-8.0	-7.2	-1.2	-5.5	-0.8
CHEMBL380230	-9.4	2.5	-6.2	-3.5	-9.7	-4.9	-9.6	1.2	-7.1	0.8
CHEMBL387326	-8.4	1.5	-5.6	-4.1	-8.2	-6.4	-8.6	0.2	-6.8	0.5
CHEMBL416956	-9.1	1.6	-6.3	-5.8	-9.1	-6.1	-9.1	-0.1	-7.1	-0.5
CHEMBL422330	-8.5	1.6	-3.9	-5.8	-8.5	-6.1	-8.3	-0.1	-5.8	-0.5

CHEMBL506	-7.0	0.1	-5.8	-3.9	-6.9	-7.7	-7.7	-0.7	-6.3	0.0
CHEMBL566534	-7.4	0.5	-5.8	-3.9	-8.2	-6.4	-7.3	-1.1	-6.4	0.1
CHEMBL682	-8.1	1.2	-5.2	-4.5	-8.3	-6.3	-7.8	-0.6	-6.1	-0.2
CHEMBL747	-7.2	0.3	-5.8	-3.9	-7.5	-7.1	-6.4	-2.0	-5.6	-0.7
CHEMBL76	-7.2	0.3	-6.3	-3.4	-6.8	-7.8	-7.1	-1.3	-5.4	-0.9

Targets	3CLV	Δ	3FNU	Δ	3K7Y	Δ	3N3M	Δ	3PHC	Δ
Crystal. Ligand.	-11.7		-9.2		-7.7		-9.6		-8.3	
CHEMBL1107	-7.1	-4.6	-7.8	-1.4	-6.9	-0.8	-3.7	-5.9	-4.2	-4.1
CHEMBL1327821	-8.4	-3.3	-8.2	-1.0	-7.4	-0.3	-5.7	-3.9	-8.7	0.4
CHEMBL1377	-7.3	-4.4	-6.4	-2.8	-7.5	-0.2	-7.6	-2.0	-8.3	0.0
CHEMBL1450	-8.3	-3.4	-8.2	-1.0	-8.9	1.2	-5.2	-4.4	-6.9	-1.4
CHEMBL1525826	-8.4	-3.3	-5.9	-3.3	-7.2	-0.5	-8.2	-1.4	-8.6	0.3
CHEMBL1535	-6.2	-5.5	-6.2	-3.0	-6.2	-1.5	-7.2	-2.4	-7.7	-0.6
CHEMBL154166	-6.9	-4.8	-7.4	-1.8	-7.4	-0.3	-7.4	-2.2	-9.1	0.8
CHEMBL1999063	-7.8	-3.9	-8.1	-1.1	-7.9	0.2	-5.2	-4.4	-8.0	-0.3
CHEMBL2104009	-6.5	-5.2	-6.5	-2.7	-6.3	-1.4	-4.8	-4.8	-7.6	-0.7
CHEMBL2110945	-6.2	-5.5	-6.5	-2.7	-7.2	-0.5	-6.1	-3.5	-6.2	-2.1
CHEMBL258608	-7.4	-4.3	-8.1	-1.1	-7.2	-0.5	-5.4	-4.2	-5.6	-2.7
CHEMBL269671	-6.0	-5.7	-7.9	-1.3	-6.4	-1.3	-6.3	-3.3	-6.2	-2.1
CHEMBL301267	-5.4	-6.3	-7.0	-2.2	-6.1	-1.6	-6.3	-3.3	-3.8	-4.5
CHEMBL303933	-7.5	-4.2	-8.8	-0.4	-8.0	0.3	-5.6	-4.0	-8.5	0.2
CHEMBL307261	-5.7	-6.0	-7.7	-1.5	-6.3	-1.4	-5.8	-3.8	-5.4	-2.9
CHEMBL339049	-7.6	-4.1	-8.6	-0.6	-7.7	0.0	-4.6	-5.0	-5.4	-2.9
CHEMBL35228	-8.1	-3.6	-8.4	-0.8	-8.0	0.3	-5.1	-4.5	-1.8	-6.5
CHEMBL36	-7.0	-4.7	-6.4	-2.8	-6.9	-0.8	-7.0	-2.6	-7.6	-0.7
CHEMBL380230	-6.6	-5.1	-9.3	0.1	-8.8	1.1	-6.2	-3.4	-9.6	1.3
CHEMBL387326	-6.8	-4.9	-7.8	-1.4	-7.0	-0.7	-5.7	-3.9	-7.6	-0.7
CHEMBL416956	-8.0	-6.1	-8.4	-1.4	-7.3	-0.7	-6.0	-5.0	-8.1	-8.3
CHEMBL422330	-5.6	-6.1	-7.8	-1.4	-7.0	-0.7	-4.6	-5.0	0.0	-8.3
CHEMBL506	-6.8	-4.9	-6.1	-3.1	-6.4	-1.3	-6.7	-2.9	-7.1	-1.2
CHEMBL566534	-6.3	-5.4	-7.8	-1.4	-7.1	-0.6	-6.0	-3.6	-2.5	-5.8
CHEMBL682	-6.5	-5.2	-7.2	-2.0	-6.8	-0.9	-5.3	-4.3	-8.3	0.0
CHEMBL747	-6.9	-4.8	-6.2	-3.0	-6.4	-1.3	-6.1	-3.5	-7.8	-0.5
CHEMBL76	-6.1	-5.6	-6.3	-2.9	-6.0	-1.7	-6.5	-3.1	-7.5	-0.8

Targets	3QS1	Δ	3T64	Δ	4B1B	Δ	4J56	Δ	4P7S	Δ
Crystal. Ligand..	-10.4		-8.1		-12.3		-13.0		-6.0	
CHEMBL1107	-8.8	-1.6	-6.2	-1.9	-8.9	-3.4	-5.3	-7.7	-6.1	0.1
CHEMBL1327821	-9.8	-0.6	-8.1	0.0	-10.1	-2.2	-9.5	-3.5	-7.0	1.0
CHEMBL1377	-7.5	-2.9	-7.2	-0.9	-8.1	-4.2	-8.1	-4.9	-5.3	-0.7
CHEMBL1450	-9.5	-0.9	-8.6	0.5	-8.4	-3.9	-9.1	-3.9	-7.0	1.0
CHEMBL1525826	-6.9	-3.5	-6.8	-1.3	-8.2	-4.1	-7.6	-5.4	-6.1	0.1

CC BY

CHEMBL1535	-6.6	-3.8	-6.5	-1.6	-7.7	-4.6	-7.7	-5.3	-5.4	-0.6
CHEMBL154166	-7.6	-2.8	-7.1	-1.0	-8.6	-3.7	-8.7	-4.3	-6.2	0.2
CHEMBL1999063	-8.5	-1.9	-6.8	-1.3	-8.8	-3.5	-9.0	-4.0	-6.6	0.6
CHEMBL2104009	-6.9	-3.5	-5.8	-2.3	-7.5	-4.8	-8.3	-4.7	-5.4	-0.6
CHEMBL2110945	-7.8	-2.6	-7.1	-1.0	-8.3	-4.0	-7.4	-5.6	-5.5	-0.5
CHEMBL258608	-7.9	-2.5	-7.7	-0.4	-7.3	-5.0	-6.9	-6.1	-6.4	0.4
CHEMBL269671	-7.4	-3.0	-7.3	-0.8	-7.0	-5.3	-7.2	-5.8	-5.7	-0.3
CHEMBL301267	-7.6	-2.8	-6.5	-1.6	-5.5	-6.8	-5.0	-8.0	-6.0	0.0
CHEMBL303933	-9.2	-1.2	-9.2	1.1	-8.5	-3.8	-9.5	-3.5	-6.6	0.6
CHEMBL307261	-7.3	-3.1	-6.8	-1.3	-6.4	-5.9	-7.5	-5.5	-5.6	-0.4
CHEMBL339049	-9.3	-1.1	-7.9	-0.2	-9.2	-3.1	-8.2	-4.8	-7.1	1.1
CHEMBL35228	-9.4	-1.0	-8.2	0.1	-6.9	-5.4	-6.1	-6.9	-6.7	0.7
CHEMBL36	-6.7	-3.7	-7.0	-1.1	-7.9	-4.4	-7.6	-5.4	-5.4	-0.6
CHEMBL380230	-8.3	-2.1	-8.5	0.4	-7.9	-4.4	-7.7	-5.3	-6.8	0.8
CHEMBL387326	-7.7	-2.7	-7.6	-0.5	-8.1	-4.2	-8.5	-4.5	-6.2	0.2
CHEMBL416956	-9.2	-1.5	-7.2	-0.6	-8.7	-8.1	-8.7	-9.8	-7.1	0.2
CHEMBL422330	-8.9	-1.5	-7.5	-0.6	-4.2	-8.1	-3.2	-9.8	-6.2	0.2
CHEMBL506	-6.1	-4.3	-6.1	-2.0	-7.4	-4.9	-7.3	-5.7	-4.9	-1.1
CHEMBL566534	-7.2	-3.2	-7.5	-0.6	-7.0	-5.3	-5.5	-7.5	-5.7	-0.3
CHEMBL682	-7.7	-2.7	-7.1	-1.0	-7.8	-4.5	-8.5	-4.5	-6.1	0.1
CHEMBL747	-6.5	-3.9	-7.2	-0.9	-7.2	-5.1	-6.8	-6.2	-5.7	-0.3
CHEMBL76	-6.7	-3.7	-6.4	-1.7	-7.8	-4.5	-7.6	-5.4	-5.3	-0.7

Targets	4QOX	Δ	PFAPT6	Δ	PFHT	Δ
Crystal. Ligand.	-8.9		-7.2		-5.7	
CHEMBL1107	-7.9	-1.0	-8.5	1.3	-7.4	1.7
CHEMBL1327821	-9.5	0.6	-9.4	2.2	-8.3	2.6
CHEMBL1377	-7.1	-1.8	-6.4	-0.8	-6.3	0.6
CHEMBL1450	-9.4	0.5	-8.4	1.2	-8.5	2.8
CHEMBL1525826	-6.8	-2.1	-6.1	-1.1	-6.4	0.7
CHEMBL1535	-6.9	-2.0	-6.6	-0.6	-6.7	1.0
CHEMBL154166	-8.0	-0.9	-7.9	0.7	-7.5	1.8
CHEMBL1999063	-8.8	-0.1	-8.3	1.1	-6.9	1.2
CHEMBL2104009	-7.1	-1.8	-6.4	-0.8	-6.2	0.5
CHEMBL2110945	-7.1	-1.8	-6.7	-0.5	-6.9	1.2
CHEMBL258608	-9.0	0.1	-8.3	1.1	-8.1	2.4
CHEMBL269671	-7.3	-1.6	-8.8	1.6	-7.3	1.6
CHEMBL301267	-6.3	-2.6	-8.0	0.8	-7.2	1.5
CHEMBL303933	-9.1	0.2	-9.3	2.1	-8.1	2.4
CHEMBL307261	-7.4	-1.5	-8.3	1.1	-7.0	1.3
CHEMBL339049	-9.3	0.4	-8.9	1.7	-8.6	2.9
CHEMBL35228	-10.2	1.3	-8.7	1.5	-9.2	3.5
CHEMBL36	-7.6	-1.3	-6.2	-1.0	-6.7	1.0

CHEMBL380230	-9.1	0.2	-8.7	1.5	-8.8	3.1
CHEMBL387326	-8.2	-0.7	-7.4	0.2	-7.6	1.9
CHEMBL416956	-8.8	-0.9	-8.1	1.3	-7.9	1.6
CHEMBL422330	-8.0	-0.9	-8.5	1.3	-7.3	1.6
CHEMBL506	-7.2	-1.7	-6.4	-0.8	-5.8	0.1
CHEMBL566534	-6.8	-2.1	-8.2	1.0	-7.3	1.6
CHEMBL682	-8.3	-0.6	-7.6	0.4	-7.2	1.5
CHEMBL747	-7.2	-1.7	-6.3	-0.9	-5.9	0.2
CHEMBL76	-7.4	-1.5	-6.3	-0.9	-6.7	1.0

Brazilian Malaria Molecular Targets (BraMMT) is available from: https://drive.google.com/open?id=1QN5zqDbpotllDmPwnyuHOYs89tInaH0V

CC BY
